# Th17 Cells in Cancer: The Ultimate Identity Crisis

**DOI:** 10.3389/fimmu.2014.00276

**Published:** 2014-06-17

**Authors:** Stefanie R. Bailey, Michelle H. Nelson, Richard A. Himes, Zihai Li, Shikhar Mehrotra, Chrystal M. Paulos

**Affiliations:** ^1^Department of Microbiology and Immunology, Medical University of South Carolina, Charleston, SC, USA; ^2^Department of Surgery, Medical University of South Carolina, Charleston, SC, USA; ^3^Department of Chemistry, College of Charleston, Charleston, SC, USA

**Keywords:** Th17, IL-17A, plasticity, immunotherapy, RORγt, cancer, tumor microenvironment

## Abstract

T helper 17 (Th17) cells play a complex and controversial role in tumor immunity and have been found to exhibit a fluctuating identity within the context of cancer. The recent, expanding literature on these cells attests to their puzzling nature, either promoting or suppressing tumor growth depending on the malignancy and course of therapeutic intervention investigated. This review addresses several newly appreciated factors that may help delineate Th17 cells’ immunological properties in the context of cancer. Several reports suggest that inflammatory signals induced in the tumor milieu regulate the functional fate and antitumor activity of Th17 cells. Recent findings also point to significant alterations in Th17 cells due to their interplay with regulatory T lymphocytes and cytotoxic CD8^+^ T cells within the tumor microenvironment. Finally, an appreciation for the stem cell-like properties of Th17 cells that augment their persistence and activity emerges from recent reports. The impact of these factors on Th17 cells’ antitumor efficacy and how these factors may be exploited to improve cancer therapies will be discussed.

## Introduction

CD4^+^ T helper 17 (Th17) cells play dynamic roles in inflammation and tumor immunity. Although, the link between inflammation and cancer has long been appreciated, researchers have just begun to elucidate the intricate – and contradictory – ways that Th17 cells insinuate themselves into this relationship. Inflammation within tumor tissue regulates immune cells (including Th17 cells) according to compelling evidence. The net effect is to dissipate antitumor immunity and contribute to the survival of cancer cells, exacerbating tumor growth and metastasis. Yet, inflammation in the presence of Th17 cells appears to initiate, maintain, and enhance protective antitumor immunity in some cases. Context may be important as the type of inflammatory response and cancer may govern whether Th17 cells display beneficial versus detrimental effects in tumor immunity. It goes without saying that understanding this process could have extraordinary clinical significance and has resulted in a rapid advance of research on Th17 cells in the field of cancer immunotherapy.

Herein, we highlight recent work that looks at tumor immunity in terms of both basic and translational aspects of Th17 cell biology. Th17 phenotype, function and their apparently mutable immunological properties will be examined. We also review the interplay between Th17 and other immune cells in malignant sites, and the mutual enhancement in antitumor activity that results from those interactions. Finally, we discuss the clinical relevance of Th17 cells in cancer therapy from the perspective of these new findings.

## Basics: T Helper Subsets in Tumor Immunity

**Figure 1:**
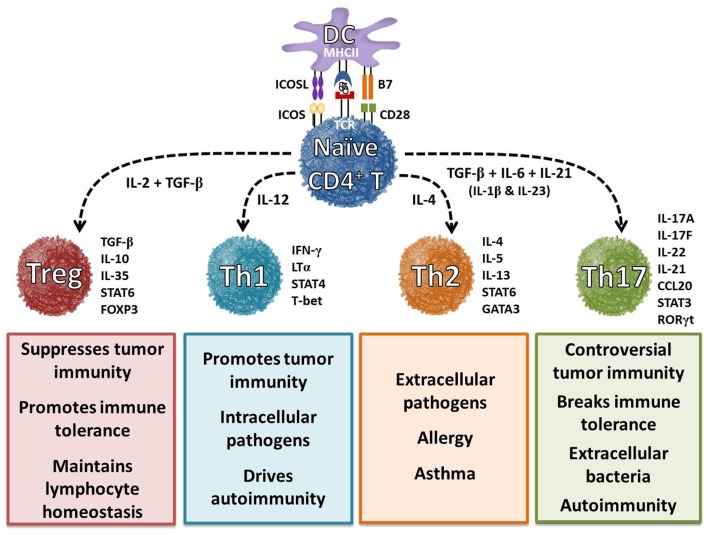
**Differentiation of helper T cell subsets is determined by cytokines**. In the presence of interleukin-6 (IL-6), IL-21, and transforming growth factor-beta (TGF-β), naïve CD4^+^ T cells differentiate into a Th17 cell phenotype, which is characterized by the expression of transcription factors retinoic acid receptor-related orphan receptor-γt (RORγt) and signal transducer and activator of transcription 3 (STAT3). IL-1β and IL-23 cytokines can promote and stabilize this phenotype during cell expansion. Once programed, these cells secrete IL-17A, IL-17F, IL-21, and IL-22, which play a key role in enhancing autoimmunity and host defense. Cytokines IL-12, IL-4, and TGF-β and transcription factors T-bet, GATA3, and FoxP3 have been shown to regulate Th1, Th2, and Treg cell development, respectively. These distinct subsets regulate immune response to foreign, self, and tumor antigens.

CD4^+^ T cells, which are key regulators of the immune system, differentiate into various T helper (Th) cell lineages with distinct biological functions (1, 2). Ultimately, CD4^+^ T cells’ ability to exert their effector functions depends on this differentiation, which arises only when professional antigen-presenting cells (e.g., dendritic cells) provide the immunological cues that prompt formation of one of several Th subsets: Th1, Th2, Th9, Th17, Th22, and FoxP3^+^ regulatory T (Treg) cells (Figure 1) (2–4). In 1989, the first two subsets of Th cells were defined – interferon-γ (IFN-γ)-producing Th1 cells that promote cell-mediated immunity and interleukin-4 (IL-4)-producing Th2 cells that support humoral immune responses (5). Despite their differences, both subsets were found to enhance antitumor immunity by inducing cytotoxic CD8^+^ T cell (CTL) expansion (6, 7). Conversely, Treg cells were found to suppress antitumor immunity by inhibiting CTLs (8, 9). Although, Th1/Th2 and Treg cells play a yin and yang role in immunity, subsequent studies found that these three lineages alone could not fully account for the development of inflammatory responses to self or tumor tissue (3, 10). A full 20 years elapsed before the knowledge gap could begin to be filled by the discovery of a third Th subset that secretes IL-17: Th17 cells (11). The identification of these cells expanded the Th1/Th2 paradigm and helped shed light on the regulatory aspects of immunity to self and tumor tissue.

T helper 17 cells’ contributions to inflammation and autoimmunity have been established without controversy, but their role in tumor immunity remains hotly debated (12–14). Some reports show that Th17 cells eradicate tumors, while other reports reveal that they promote tumor progression. A satisfying, all-encompassing explanation for these conflicting results has not been forthcoming. However, recent work has provided a clue: Th17 form and function are uniquely sensitive to a host of factors, including the type of cancer (e.g., prostate versus pancreatic), the therapeutic approach (e.g., vaccine versus adoptive cell transfer therapy) and the stimuli to which the cells are exposed during activation (e.g., T cell receptor strength). Thus, understanding the cytokines and transcription factors that regulate Th17 cell responses in the tumor milieu will be critical for advancing efficacious cancer therapies.

## Differentiation and Function of Th17 Cells

T helper 17 cells represent a CD4^+^ lineage distinct from Th1, Th2, and Treg cells and are characterized by a unique molecular and functional signature (15). Naïve CD4^+^ T cells undergo differentiation into specific Th subsets via specific cytokine signals (Figure 1). Th17 cells develop from naïve CD4^+^ T cells in the presence of TGF-β, IL-6, and IL-1β and are maintained long-term in the presence of IL-21 and IL-23 (16). Th17 cells are characterized by their capacity to secrete IL-17A, IL-17F, IL-21 IL-22, and CCL20 (17–19). Additionally, Th17 generation is controlled by the master transcription factors retinoic acid-related orphan receptor (ROR)γt, RORα, aryl hydrocarbon receptor (AHR), and interferon regulatory factor 4 (IRF4) (20–24). Cytokines and transcription factors produced by Th17 cells can have both beneficial and pathogenic effects. These controversial findings are further complicated by direct environmental effects on Th17 differentiation and function.

Environmental factors, such as toxins and ultraviolet light, have recently been reported to play a role in the survival and function of Th17 cells. As previously mentioned, the ligand-dependent AHR transcription factor helps drive the differentiation of Th17 cells (25). Ligands to activate AHR include hydrocarbons, such as dioxins, which are toxic chemical compounds (26). Studies have shown that the binding of these toxins, as well as other dietary compounds, to AHR results in its activation and can drive the differentiation of Th17 cells (22). Specifically, ligation of dioxins to AHR increases the production of IL-22, IL-17A, and IL-17F by Th17 cells and can lead to exacerbated autoimmunity. In addition to environmental toxins, it has been reported that exposure to ultraviolet light can also affect the Th17 milieu. Phototherapies using UV light have shown success in the treatment of exacerbating skin diseases such as psoriasis and atopic dermatitis (27, 28). Extensive analysis by Furuhashi et al. revealed that psoriasis patients treated with UV therapy had reduced skin lesions in coordination with decreased levels of Th17 cells. Furthermore, responders and non-responders could be predicted based on the expression of Th17 cells prior to treatment, with increased levels favoring a poor response. Building on previous findings that UV treatment reduces the levels of IL-17 and IL-22 in psoriasis patients (29), these findings indicate that Th17 cells can be regulated by the environment. However, caution is warranted based on more previous findings that UV treatment is an effective local treatment, but does not augment Th17 cytokines systemically (30). Collectively, these studies show that both localized (i.e., cytokines) and environmental (i.e., toxins and UV exposure) factors can affect Th17 cells and their resulting autoimmune manifestations. Due to the dual nature of Th17 cells in both cancer and autoimmune disease, it’s important to identify the presence of these cells in various diseases. In addition to the previously mentioned identification markers, Th17 cells can also be delineated by the increased presence of dipeptidyl peptidase IV, called CD26, on their cell surface.

CD26 is a multifunctional ectoenzyme involved in multiple facets of T cell activation and function (31). Interestingly, T cells with the highest expression of CD26 secrete the greatest amount of IL-17A, which is the hallmark cytokine of Th17 cells. In contrast to Th17 cells, Treg cells express low levels of CD26 and high levels of the ectonucleotidases CD39 and CD73 (32). Moreover, CD26 up-regulation correlates with disease activity in human autoimmune manifestations linked to the presence of pathogenic Th17 cells, such as rheumatoid arthritis (31) and diabetes (33). Elevated CD26 expression – as well as high expression of the inducible costimulator (ICOS), the IL-23 receptor (IL-23R), and chemokine receptor 6 (CCR6) – distinguishes Th17 cells from other human T cell subsets (34, 35). Furthermore, the authors reported that the expression of extracellular CCR4, CCR6, and CXCR3 can be used to identify human Th1, Th2, and Th17 cells in healthy and diseased individuals. Indeed, the identification of CD4^+^ T cell subsets via these various extracellular markers has helped investigators shed light on developmental and/or functional relationships between Th17 and other T cells subsets in cancer and infectious disease.

## Distinctive Features of Classical and Non-Classical (Th17-Derived) Th1 Cells

**Figure 2:**
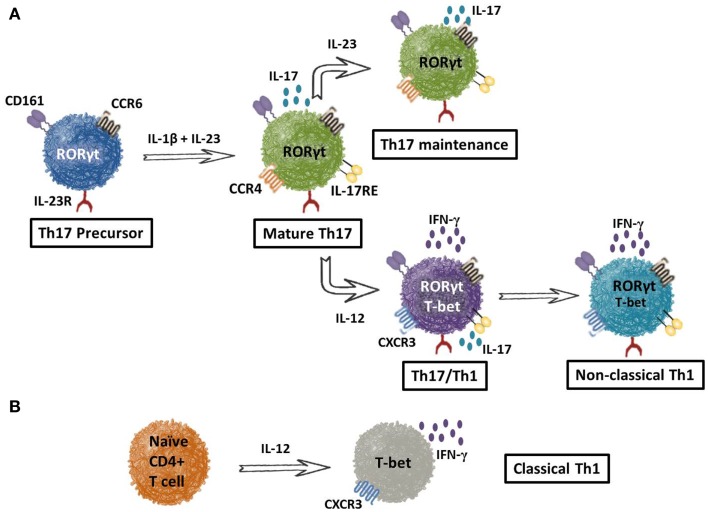
**Culture conditions drive the expression of Th17 or non-classical Th1 cells**. Cell surface receptors distinguish T helper subsets. **(A)** Th17 precursors can be identified from peripheral blood by the presence of lectin-like receptor CD161, chemokine receptor 6 (CCR6), and IL-23 receptor (IL-23R). In the presence of IL-1β and IL-23, these precursors differentiate into mature Th17 cells capable of IL-17A production and identified by the addition of CCR4 and IL-17 receptor E to their surface repertoire. In the presence of IL-23, the Th17 phenotype is maintained as seen with the preservation of all surface markers, IL-17A and RORγt. When cultured with IL-12, however, mature Th17 cells adopt a divergent phenotype that has both Th1 and Th17-like aspects. These cells, termed Th1/Th17 cells, express CXCR3 instead of CCR4, produce both IFN-γ as well as IL-17A, and have a high expression of RORγt with an intermediate expression of the Th1 transcription factor, T-bet. In the continued presence of IL-12, Th1/Th17 cells can adopt a Th1-like phenotype and are termed non-classical Th1 cells. As shown, non-classical Th1 cells have an increased expression of T-bet, decreased RORγt, and produce only IFN-γ. **(B)** Classical Th1 cells are directly derived from naïve CD4^+^ T cells in the presence of IL-12 and can be identified through the expression of CXCR3, IFN-γ, and T-bet.

Although cell surface markers provide a way to identify Th17 cells from other subsets, the recent finding that Th17 cells can convert into the Th1 lineage (gain an ability to secrete IFN-γ and lose their capacity to secrete IL-17) – a phenomenon referred to as “plasticity” (36) – has complicated our ability to discriminate these cells in the tumor-bearing host. Thus, the question of how to distinguish non-classical Th1 cells (i.e., Th17s that have converted to Th1) from classical Th1 cells arises. Recent work suggests that the surface marker lectin-like receptor CD161 discerns these two subsets. As shown in Figure 2A, Th17 precursors can be detected by CD161 in cord blood, as these cells do not yet express IL-17A at the mRNA or protein level (37). Along with CD161, Th17 precursors express IL-23R and CCR6. When exposed to IL-1β and IL-23, precursors transition into mature Th17 cells with the ability to produce IL-17A. Conversely, when Th17 cells encounter IL-12, they convert to a Th17/Th1 phenotype that co-expresses RORγ, T-bet, CXCR3, CCR6, CD161, and IL-23R. Continued IL-12 presence (or comparable signals) converts Th17/Th1 cells into a Th1-like phenotype. These ex-Th17 cells are known as non-classical Th1 cells due to their sustained expression of CD161. In contrast, classical Th1 cells do not arise from Th17 precursors and thus, do not express CD161. Rather, classical Th1 cells manifest from naïve CD4^+^ T cells in the presence of IL-12 (Figure 2B). Very recent work has also implicated the transcription factors Runx1 or Runx3, in combination with T-bet, to be crucial for the generation of IFN-γ-producing Th17 cells (38). Indeed, additional investigations to determine the impact of non-classical versus classical Th1 cells in tumor immunity will be important in designing therapies for patients with cancer.

## Distribution of Th17 Cells and Their Differential Impact on Tumor Immunity

While Th17 cells are abundant in the mucosal tissues and support gut-related homeostasis, few Th17 cells (~0.1%) reside in the peripheral blood of healthy individuals or cancer patients (13, 39, 40). However, a significantly greater number of Th17 cells infiltrate tumors, especially compared to the density of Th17 cells in the adjacent, non-tumor tissue of patients. This heightened presence of Th17 cells in tumor tissue holds true for a vast range of malignancies, implying that tumors themselves produce factors that promote Th17 cell trafficking to the diseased site (41–59).

Gut microbes also produce factors that promote Th17-mediated tumor growth. A commensal bacterium called enterotoxigenic *Bacteroides fragilis* (ETBF) was recently reported to induce Th17 cells and to play a role in promoting colon carcinogenesis (60). Moreover, Th17 cells were found to directly promote tumor growth, as neutralization of IL-17 and IL-23R reduced the number of tumors that developed in the distal colon of mice. Recent studies from Wick et al. have shown that induction of the Th17 immune response by ETBF appears to hinge on Stat3 activation in immune cells (61). Given this new finding, two mechanisms can be targeted to reduce tumor growth by ETBF: one, gut microbes can be therapeutically targeted with antibiotics and two, the long-term activation of Stat3 can be inhibited to decrease a Th17 immune response. Interestingly, however, gut microbes perturbed by lymphodepletion/chemotherapy, a phenomenon called microbial translocation, *improve* adoptive CD8^+^ T cell immunotherapies for melanoma (62). Yet, how the induction of Th17 cells by microbial translocation impacts cell-based therapies for various cancers remains unknown and will be important for creating future treatments.

The high frequency of Th17 cells that exist in tumors (47) permits researchers to examine their capacity to either promote or suppress tumor growth. However, such work has only added to the confusion concerning Th17 cells in cancer. Pro-inflammatory cytokines secreted by Th17 cells, such as IL-17A, impair immune surveillance and promote tumor growth (63, 64). Conversely, Th17 cells have been reported to directly eradicate melanoma tumors in mice to a greater extent than Th1 cells (65, 66). Those studies involved an adoptive T cell transfer (ACT) therapy approach, which takes advantage of CD4^+^ T cells that express a TCR recognizing tyrosinase tumor antigen (65). Exploitation of the TCR leads to rapid expansion of Th17 populations to large numbers *ex vivo* for reinfusion into the autologous tumor-bearing hosts. This approach parallels ACT trials in human patients and has allowed investigators to examine how infused TCR-specific Th17 cells interact with other immune cells in the body. These interactions may either enhance or impair treatment outcome and could hold the key to understanding the Janus-faced effects of either pro- or antitumor Th17 cells. The interactions of Th17 cells and host immune cells will be discussed later in this review, but first a better understanding of the controversial roles of Th17 cells in cancer is discussed directly below.

## Yin and Yang of Inflammatory Th17 Cells in Tumor Immunity

The suspected relationship between inflammation and cancer began more than a century ago, but researchers today are still unraveling the importance of this affiliation in tumor progression (67–71). Depending on the type of cancer encountered, a number of factors could alter the effect of Th17 cells on a malignancy’s pathology, including: the source of the Th17 cells (arising naturally via tumor growth or adoptively transferred following *ex vivo* manipulation), the functional phenotype of the cells and/or exposure to therapeutic interventions such as chemotherapy. Understanding how Th17 cells cause inflammation in the context of these factors, as well as how these elements impact patient survival, is of considerable interest in the field of oncology. One thing that remains clear is that the influence of Th17 cell accumulation in tumors on cancer progression is controversial. Some small measure of consensus is arising from the controversy: Th17 cell subsets can possess either regulatory or inflammatory properties depending on the stimuli they encounter. These divergent phenotypes may explain why Th17 cells have potent antitumor properties in some experimental regimens but actually foster tumor growth in others.

One possible explanation for this controversial phenomenon could be that different types of tumor tissue foster the generation of Th17 cells with different phenotypes. The generation of Th17 cells with opposing phenotypes in response to different tumor tissue milieus would satisfyingly resolve the experimental discrepancies. Indeed, high frequency Th17 cell infiltration into the tumor bed of patients with colon or pancreatic cancer strongly correlates with poor prognosis (72, 73). Conversely, increased Th17 cell numbers in ovarian tumors have been associated with improved patient survival rates (74–78). How the tumor regulates downstream signaling pathways in Th17 cells might also impact their fate, as Kim and coworkers found that natural versus induced Th17 cells are regulated differently by Akt and mTOR pathways (79). Identification of the tumor-localized triggers that shape distinct Th17 cell responses will be invaluable for progress in the cancer immunotherapy field. An obvious direction is to identify the antigen-specificity of tumor-infiltrating Th17 cells. Very possibly, the Th17 cells with different antigen-specificity might have different impacts on the clinical outcome of cancer. Despite the vast number of unknowns, one obvious role of Th17 cells in tumor progression is their contribution to local inflammation.

## Th17 Cell-Mediated Inflammation in Cancer

Inflammation has long been recognized as a mediator of tumor progression in diseases such as colorectal (80), pancreatic (81, 82), and lung (83) cancer. This phenomenon is largely based on the continuous cell proliferation and cytokine production occurring at sites of inflammation. More recent studies have shown that alternative factors, such as β-catenin, can further augment local T cells to increase pro-inflammatory cytokine production. Keerthivasan et al. showed that activation of the Wnt/β-catenin signaling in both Th17 and Treg cells correlates with the progression of colitis and colon cancer (84). Not only did tumor growth correlate with enhanced pro-inflammatory cytokines, but these findings could be reversed in RORγ^−/−^ mice. Given that Th17 cells are dependent on RORγ, this finding is important for elucidating how these cells drive tumor progression. Furthermore, protein levels of IL-1β, IL-21, and TGF-β have been found to be up-regulated in patients with gastric cancer (85). Based on the role of these cytokines in Th17 differentiation, the microenvironment of these patients appears to be more conducive for Th17 cell expansion. The subsequent growth and cytokine production (IFN-γ and IL-17) from these cells drives further inflammation and cancer cell growth. Although these cytokines can drive inflammation-dependent tumor growth, they also play controversial roles in cancer progression or regression as discussed below.

## Th17 Cells and Tumor-Associated Angiogenesis

Inflammatory Th17 cells and their associated cytokines (i.e., IL-17A, IL-17F, IL-21, IL-22, etc.) mediate tumor growth in two distinct ways – by driving angiogenesis and by suppressing antitumor immunity (86). Among the cytokines secreted by Th17 cells, IL-17A is best known to induce angiogenesis in tumor tissue. Angiogenesis facilitates tumor growth by providing the malignancy with a migratory egress to healthy tissues in patients. Interestingly, tumors transfected with IL-17A were found to grow and vascularize more rapidly than wild-type tumors in mice. Conversely, genetic IL-17A ablation impaired the growth of tumors in mice (87). Positive correlations between the density of tumor-infiltrating Th17 cells and increased micro-vessels have been reported in many human cancers. Further work by Chang et al. has shown that the production of IL-17A by Th17 cells also results in the recruitment of myeloid suppressor cells (88). Depletion of IL-17A or myeloid suppressor cells resulted in tumor reduction *in vivo*. These findings provide an angiogenesis-independent mechanism by which IL-17A promotes tumor growth. Collectively, these data suggest that IL-17A-producing T cells promote tumor progression via multiple mechanisms (13, 89, 90).

Yet, other cytokines secreted by Th17 cells (IL-17F, IL-21, and IL-22) exhibit anti-angiogenic properties, convoluting the overall correlation between Th17 cell activity and tumor growth in the context of angiogenesis (91–93). The conditions that prompt Th17 cells to secrete one or more of these cytokines may regulate angiogenesis. Moreover, the critical setting of the type of tumor that Th17 cells encounter could have some bearing on the outcome of their regulatory role. In light of the findings by Sallusto’s group that different pathogens promote the generation of either effector or regulatory Th17 cells (94), it is possible that different types of cancers will induce Th17 cells that can either facilitate or suppress angiogenesis by differentially regulating IL-17A, IL-17F, IL-21, and IL-22 secretion in patients. For example, the heightened production of IL-22 by Th17/Th22 cells in patients with pancreatic (95) or lung (96) cancer correlates with poor prognosis and survival. On the contrary, IL-22 has been shown to mediate tumor reduction in certain models of breast cancer (92). Unraveling the regulatory patterns of Th17 cells may require a deeper investigation into the plethora of cytokines within the tumor milieu of a variety of cancers.

## Immunosuppressive Properties of Th17 Cells

**Figure 3:**
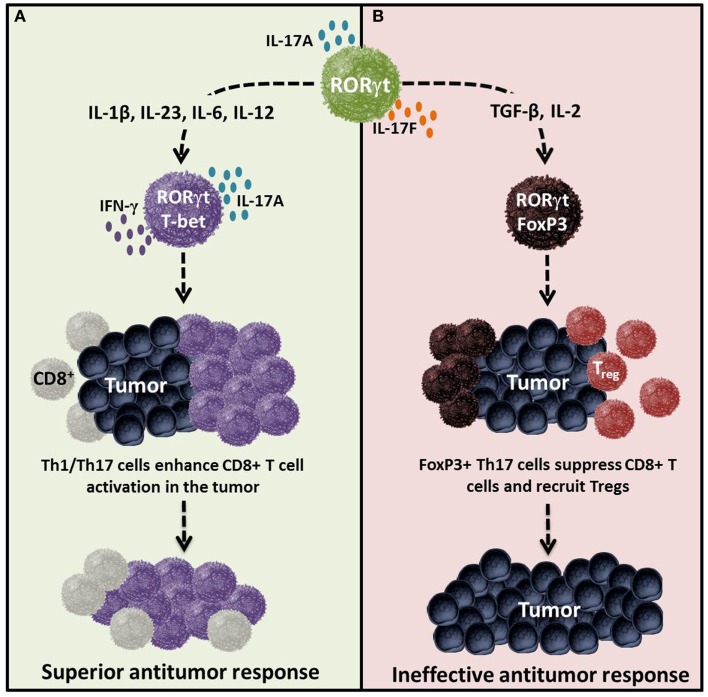
**Cytokines determine the effector versus regulatory nature of Th17 cells in tumor immunity**. Cytokines and costimulatory molecules distinctly transform Th17 cells into either an effector or regulatory phenotype, which in turn regulates immunity to self/tumor tissue. **(A)** Effector Th17 cells activated with IL-1β, IL-23, IL-6, IL-12 and/or ICOS agonist are poly-functional and are capable of mediating potent antitumor immunity. **(B)** Regulatory Th17 cells programed with cytokines such as TGF-β, IL-2, and/or CTLA4 can dampen their function and persistence, thereby potentially reducing their capacity to kill tumors. Regulatory Th17 cells likely do not foster the induction or cooperation of CTLs to the malignant site.

**Figure 4:**
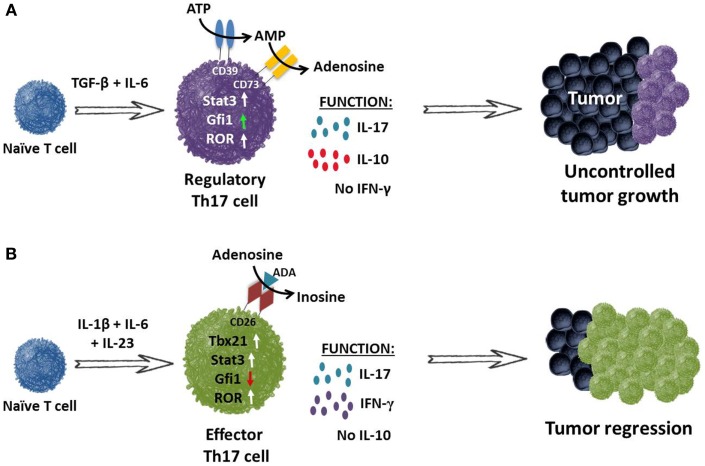
**TGF-β induces Th17 cells to express ectonucleotidases and release immunosuppressive adenosine. (A)** Th17 cells programed with TGF-β and IL-6 fail to secrete IFN-γ but do secrete IL-17A and IL-10. These cells expressed nominal amounts of Gfi1 (growth factor independent protein 1 – repressor of ectonucleotidase), resulting in CD39 and CD73 ectonucleotidase expression on their cell surface. CD39 and CD73 convert ATP to immunosuppressive adenosine, thereby contributing to the inhibition of antitumor immunity. **(B)** Conversely, programing CD4^+^ T cells in the absence of TGF-β but presence of IL-1β, IL-6 and IL-23 supports the generation of Th17 cells that secrete IL-17A and express RORγt and STAT3. Moreover, these cells also express transcription factor Tbx21 and co-secrete IL-17 and IFN-γ but not the immunosuppressive cytokine IL-10. These inflammatory Th17 cells also express increased Gfi1. It is also possible that they express CD26, which facilitates the conversion of adenosine to inosine upon binding of adenosine deaminase (ADA). These infused cells promote the activation of CD8^+^ effector T cells and cooperate to mediate tumor regression in an IFN-γ and IL-17A-dependent manner.

Although Th17 cells eradicate tumors when transferred into mice, they also function as regulatory cells with the capacity to suppress antitumor immunity (12). Two distinct mechanisms that sustain their immunosuppressive nature have been identified. One, Th17 cells are capable of converting into Treg cells (i.e., plasticity; Figure 3) (97, 98); two, Th17 cells release immunosuppressive adenosine upon TGF-β-dependent ectonucleotidase expression (99) as illustrated in Figure 4 and described in detail below.

### Th17–Treg plasticity promotes tumor suppression

In contrast to classical Th1 cells, Treg and Th17 cells convert into other lineages. Th17 cells may originate from Treg cells with differentiation mediated by IL-1β interaction with Tregs expressing the IL-1 receptor (IL-1R) (38, 100–102). Th17 cells can also undergo lineage conversion into Tregs, indicating that plasticity is a two-way street. Astoundingly, this cellular inter-conversion does not have rigid binary outcomes: *intermediate* phenotypes that co-express FoxP3 and RORγt may also arise (103). These hybrids display immunosuppressive functions toward CD8^+^ T cells (104). Hence, distinguishing this discrete population from bona fide subsets will be critical for a clear elucidation of the regulation of tumor immunity by Th17 cells.

### TGF-β induces Th17 cells to express ectonucleotidases

Cytokines TGF-β, IL-6, and IL-23 program naïve CD4^+^ T cells toward a Th17 phenotype and have been reported to enhance autoimmune manifestations, particularly autoimmune encephalomyelitis (EAE) (105). To test the role of these cytokines in EAE, Th17 cells were generated in the presence of TGF-β plus IL-6 or IL-23. As expected, cell subsets from both cultures secreted IL-17A, but interestingly, only IL-23-cultured cells induced pathologic lesions in EAE mice. The authors postulated that the combination of TGF-β and IL-6 imprints Th17 cells with an immunosuppressive phenotype. The finding that TGF-β/IL-6-cultured cells produced IL-10 upon myelin antigen recognition supported this idea, as IL-10 dampens immune responses to self-antigen.

Guided by the autoimmune results in EAE, Chalmin et al. postulated that Th17 cells programed with IL-23 (plus IL-1β and/or IL-6) will eradicate tumors when transferred into mice, while those programed with TGF-β and IL-6 will be less effective (Figure 4) (99, 106). Mechanistic studies revealed that TGF-β/IL-6-cultured Th17 cells co-express CD39 and CD73 ectonucleotidases on their surface. Concomitant expression of these two enzymes transforms ATP or ADP into immunosuppressive adenosine (99). These signaling events impair the antitumor activity of Th17 cells. As seen in Figure 4A, TGF-β and IL-6 induce CD39 and CD73 expression on Th17 cells by decreasing growth factor independent protein 1 (Gfi1) and activating STAT3, resulting in IL-17 and IL-10 secretion.

In contrast, Th17 cells programed with IL-1β, IL-6, and IL-23 do not express CD39 or CD73 (Figure 4B). These cells co-expressed T-bet and RORγt, resulting in secretion of IFN-γ and IL-17, but not IL-10. Moreover, these Th17 cells mediated robust tumor regression in mice, as reported by several labs (99, 107). Thus, changes to the cytokines used in generating Th17 cells can drastically impact the cell-mediated responses to tumors. While the cytokines used to expand Th17 cells are instrumental for translational therapies, it is also clear that the generation of Th17 cells with durable memory to tumors is even more critical, particularly for gene-modified T cells for cancer. Fortunately, one consistent finding has been the necessity of IL-23 for the maintenance of Th17 cells.

Further work by Kuchroo’s group has shown that the signaling induced by the interaction of IL-23 with IL-23R on Th17 cells is crucial to obtain a pathogenic and sustained phenotype (108). These authors discovered an essential downstream kinase, called serum glucocorticoid kinase-1 (SGK1), which regulates IL-23R expression on the cell surface. The addition of low levels of TGF-β1 was sufficient to up-regulate IL-23R in Th17 cells and to subsequently sustain their differentiated phenotype. Further studies revealed that this finding mechanistically hinged upon the deactivation of Foxo1, which appears to function as a Th17 suppressor, following IL-23R induction. Intriguingly, SGK1 regulates salt homeostasis and is also able to up-regulate IL-23R following an increase in salt concentration, which could partially explain the rise in autoimmune diseases in today’s society. These findings indicate that maintenance and pathogenicity of Th17 cells relies heavily on the binding of IL-23 to IL-23R.

Additional studies of the IL-23R in Th17 cell maintenance revealed a striking difference when cultured with the cytokines TGF-β1 or TGF-β3. While these cytokines signal through the same receptor in Th17 differentiation, the addition of TGF-β3 to the cell culture resulted in an enhanced expression of *Il22* and *Il23r* (109). Furthermore, these cells were more pathogenic and induced severe EAE in mice compared to those given TGF-β1. Experimentally, cytokines TGF-β, IL-6, IL-21, IL-23, and IL-1β have been used in different combinations by a number of groups to generate Th17 cells. The ramification of these results is that these cytokines cannot be used interchangeably to generate Th17 cells as they have differential effects on the phenotype.

## Stem Cell-Like Memory Th17 Cells in Tumor Immunity

Gene therapy enables researchers to engineer T cells with TCR or chimeric antigen receptors (CAR) that recognize tumor antigen, which has unlocked new ACT treatments of unprecedented efficacy (110–112). Unfortunately, most clinical trials have not risen to their therapeutic expectations, marred by the use of terminally differentiated T cells (113). Thus, a need arises for the generation of memory T cells. Th17 cells display durable persistence and the ability to mount rapid recall responses to tumors (107). Yet, effective means of generating Th17 cells with memory are still unclear. Whereas much of our understanding of memory has been gleaned from studies of CD8^+^ T cells, recent advancements in defining the features of memory CD4^+^ T cells have trickled into the literature.

CD4^+^ T cells differentiate into distinct subsets upon antigenic encounter, adding complexity to the issue of memory (114). Further complicating the matter, Th17 plasticity in their late developmental programing permits them to acquire at least some Th1 or Treg-like features. This plasticity is likely due to the prevailing cytokines and signaling strength that they receive during recall (36). Th17 diversity poses unique challenges to conclusively defining memory phenotype, which makes it difficult to discern if the mechanisms that maintain hematopoietic stem cells (HSC) self-renewal are functional in Th17 cells.

**Figure 5:**
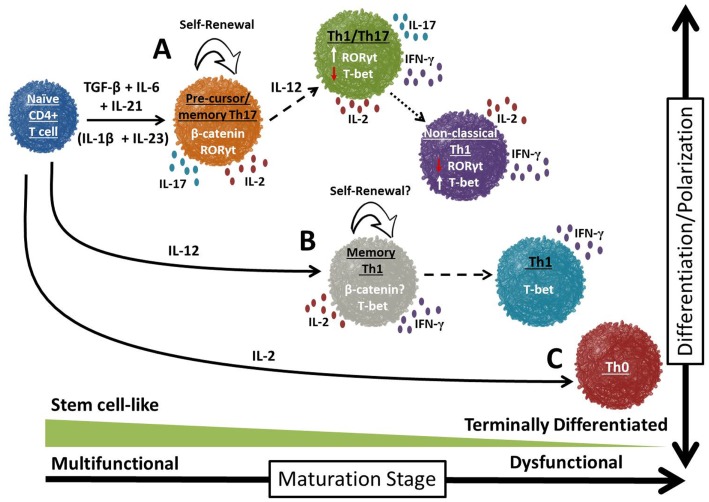
**Divergent potential for self-renewal and plasticity in T cell subsets**. Over time, the majority of CD4^+^ T cells differentiate into terminally differentiated effectors that possess short-lived immune responses to tumor antigen. However, a small proportion of these cells will enter into a self-renewing pool, resulting in the generation of long-lived memory T cells. Th17 cells appear to exist in a progenitor-like stage (**A**; Th17 precursor) compared to Th1 or Th0 cells (**B** and **C**, respectively). Th17 cells possess a number of uniquely exciting traits: enhanced self-renewal, multi-functionality and rapid recall responses to tumor antigen.

Murine Th17 cells were recently discovered to be long-lived, to possess a high proliferative potential upon antigenic re-encounter and to self-renew with enhanced poly-functionality *in vivo* compared to their Th1 counterparts (Figure 5) (107). These data were unexpected given that Th17 cells express extracellular markers of terminally differentiated effector memory *in vitro* (e.g., low CD62L and CCR7 levels; high CD44 levels). Yet, these cells were camouflaged as terminal cells *in vitro*; once infused, the cells resumed CD62L and CCR7 expression, indicative of a less differentiated phenotype. Several pathways expressed in memory T cells were identified as operational in these cells. For example, Th17 cells expressed *Lef1* and *Tcf7* (downstream genes in the Wnt/β-catenin pathway) to a greater extent than Th1 cells. This pathway is critical for the generation of HSCs and has been found in stem cell-like CD8^+^ T cells (115). *In vivo*, Th17 cells not only gave rise to Th1-like progeny, but also possessed a self-renewing capacity. Dual-function was required for Th17 cell-mediated tumor destruction because cells deficient in IFN-γ or IL-17A had impaired activity. Thus, the short lifespan of *in vitro* Th17 cells proves deceptive.

Mirroring results from murine Th17 cells, human Th17 cells display durable memory to antigen in a variety of diseases (116). Specifically, human Th17 cells were studied in the pathological microenvironments of graft-versus-host disease, ulcerative colitis, and cancers. The number of durable memory Th17 cells was increased in the chronic phase of these diseases. When transferred into xenograft models, human Th17 cells mediated antitumor immunity and had a high capacity to persist *in vivo*. These cells expressed a relatively specific gene signature that incorporated abundant anti-apoptotic genes and were resistant to activation-induced cell death due to high c-FLIP expression (117). Together, these data indicate that human Th17 cells exhibit the hallmark properties of memory T cells, genetically similar to those found in HSCs. The Th17 pathways associated with memory response thus present themselves as attractive targets for manipulation, as controlled activation of these pathways may lead to therapeutic advances. Furthermore, predicting how these cells will interact with host immune cells is important for therapeutic efficacy and is discussed directly below.

## Th17/Immune Cell Interplay in the Tumor

### Th17-CD8 dynamics

It has recently been discovered that Th17 cells increase the function and frequency of CD8^+^ T cells in the tumor. Specifically, adoptively transferred Th17 cells have been reported to activate endogenous CD8^+^ T cells in mice with melanoma, which was crucial for the antitumor effect (66). These studies revealed that Th17 cells promoted dendritic cell recruitment into the tumors, thus inducing CTL expansion. Th17 cells also promoted CCL20 chemokine production by tumor tissues, thereby recruiting CD8^+^ T cells to the malignant site. More recent work by Munegowda et al. has shown that Th17 cells can activate CD8^+^ T cells in the tumor milieu in a variety of ways, utilizing both direct and indirect mechanisms (118). First, Th17 cells can directly interact with CD8^+^ T cells via the acquisition of major histocompatibility complex/peptide (pMHCI) and is crucial for CD8^+^ T cell response. Second, soluble factors released by Th17 cells, such as IL-2 but not IL-17, aid in the activation of CD8^+^ T cells. Finally, Th17 cells that have homed to the tumor – due to their vast chemokine expression – can stimulate tumor tissue to produce chemoattractants (i.e., CCL20), which then recruit CTLs to the tumor (66).

A potential synergistic interaction between Th17 and CD8^+^ T cells emerges from these antitumor studies, but work from the Antony lab suggests caution against overemphasizing this interplay. The authors reported that donor CD4^+^ T cells eradicate tumors directly without the need for host CD8^+^ T or NK cells (119). These contrasting results highlight the need for follow up studies on the role of antitumor CD4^+^ T cells (as well as Th1 and Th17 cells) on host or infused CD8^+^ T cells. What remains clear, however, is that Th17 cells – under the right conditions – can mediate tumor regression in mice with melanoma. Interactions between Th17 and CD8^+^ T cells may have certain consequences for the treatment outcome; however, another important question, with ramifications for the efficacy and persistence of these treatments, concerns the proportion and effects of Th17 and Treg cells on each other and on tumor regression.

### Th17–Treg dynamics

The influence of Tregs on Th17 cells remains incompletely elucidated, though the potential role of Tregs in dampening antitumor responses is known. One particular focus on Th17–Treg interactions in immunotherapy involves the effect of IL-2 – often administered to mice in ACT experiments to support the expansion of infused Th17 cells. IL-2 signaling exerts significant, yet divergent, regulatory effects on Th17 and Treg cells in the tumor (120). Thus, IL-2 bolsters Th17 cells, which subsequently dampen host Tregs in the tumor. These findings would suggest that infused Th17 cells reduce the number of host Tregs. Subsequent abrogation of Treg suppression of tumor immunity offers one explanation for why the therapeutic outcome in these Th17-based ACT treated mice is often curative.

Alternate explanations for conflicting data in the literature may be preferred. For example, Treg cells require IL-2 to overcome FoxP3-mediated apoptotic properties for their *in vivo* maintenance (121) and out-compete other subsets (i.e., Th17) for the molecule via a high affinity IL-2 receptor. Thus, it is conceivable that Tregs impair Th17 engraftment by depriving them of IL-2, a situation that would certainly hamper antitumor immunity. On the other hand, given that high IL-2 concentrations impair Th17 expansion and function, Treg cells may actually *support* the engraftment and function of Th17 cells by functioning as an IL-2 cytokine sink (122), as was reported by the McGeachy lab. If so, host Treg depletion would impair the persistence of antitumor Th17 cells.

**Figure 6:**
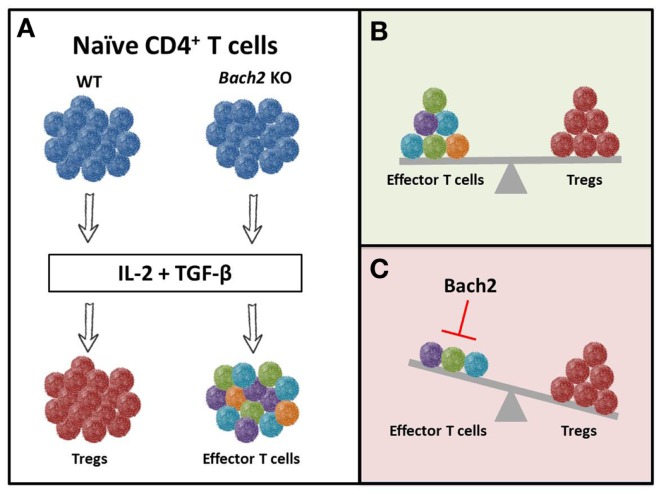
***Bach2* inhibits T-effector differentiation and stabilizes Tregs**. Cytokines present in the environment regulate the phenotypic and functional fate of T cells. The combination of IL-2 and TGF-β creates an environment conducive for the generation of inducible Treg cells. **(A)** WT cells differentiated into FoxP3^+^ Treg cells when cultured with these cytokines, while *Bach2* knockout cells converted into effector T cells. This suggests that *Bach2* is **(B)** an instrumental regulator of the immune system through **(C)** inhibition of effector function and thereby, stabilization of Tregs.

Restifo and colleagues very recently reported a novel role of the transcriptional repressor BACH2 in regulating Treg differentiation and decreasing the effector function of Th1 and Th17 cells (123). BACH2 is a known regulator of Blimp-1 in B cells required for class switch recombination; however, its role in T cells was unknown (124). When CD4^+^ T cells were programed toward an inducible Treg subset (via IL-2 and TGF-β), wild-type cells converted to FoxP3^+^ Treg cells while *Bach2* KO cells differentiated into effector T cells (Figure 6A). Since a balance between regulatory and effector cells is needed to maintain homeostasis (Figure 6B), BACH2 serves as a critical regulator of the immune system. Interestingly, *Bach2* deficient cells exhibited superior effector function as they secreted heightened levels of IL-17, IFN-γ, or IL-13 when programed toward a Th17, Th1, or Th2 phenotype, respectively (123). The authors also found that BACH2 not only stabilizes Treg formation, but also blocks the generation of effector T cells (Figure 6C). Thus, manipulating BACH2 expression in T cells could bolster vaccine or cell-based immunotherapies for cancer.

## Cancer Therapies: Employing the Dual Nature of Th17 Cells

The number of T cell-based therapies available for cancer patients has grown exponentially in recent years. As shown in this review, the mere presence of Th17 cells does not correlate with a unified prognosis and could result in increased or decreased tumor burden. For patients tumors that are exacerbated by Th17 cells (i.e., pancreatic), an obvious therapeutic target is to reduce the number of these cells in the tumor microenvironment. Very recent work has shown that treating pancreatic tumor-bearing mice with certain compounds, such as Embelin (XIAP inhibitor), can alter the tumor microenvironment by skewing CD4^+^ T cells away from Th17 differentiation and toward a Th1 phenotype (125). Among several changes seen in the tumor microenvironment following Embelin administration, the levels of IL-6/STAT3 were significantly decreased and resulted in a reduced population of Th17 cells. Overall, this helped reduce Th17-mediated inflammation within the tumor and prevented further growth of the malignant cells. Previous work by Chang et al. showed that the production of IL-17 by Th17 cells in the mouse lung cancer model K-ras (G12D) was sufficient to drive tumor growth through the recruitment of myeloid suppressor cells (88). Upon inhibition of IL-17, the mice exhibited tumor reduction that resulted from decreased tumor cell proliferation and angiogenesis. Thus, the specific targeting of Th17 cells or their related cytokines could be promising for patients with enhanced Th17-based inflammation in the tumor microenvironment.

On the contrary, clinical trials to boost specific T cell populations via ACT have shown great promise in treating cancer patients. In these trials, lymphocytes [either tumor-infiltrating lymphocytes (TIL) or gene-engineered PBL] are often expanded with high dose IL-2 (6000 IU/ml) and soluble OKT3 (anti-CD3), or are expanded with magnetic beads decorated with CD3 and CD28 agonists (126, 127). Intriguingly, work by Paulos and coworkers suggest that new methods of expanding human T cells to a Th17 phenotype could be promising for ACT therapy. This group discovered that activation of human Th17 cells with CD3 and ICOS agonists enhanced their activity when transferred into tumor-bearing mice compared to those activated with CD28 (128). Despite these findings, Th17 cells and ICOS ligation have yet to be exploited in the clinic (129). However, gene therapy now permits the opportunity to redirect Th17 cells (via TCR or CAR that recognize tumor antigen) for the potential treatment of a broader range of malignancies (130–135). This approach could circumvent the use of inefficacious differentiated T cells obtained from TIL (113, 136). Given the significant antitumor response seen following the infusion of Th17 cells into mice bearing certain cancers (i.e., melanoma), future work to translate and redirect these cells to eradicate tumor tissue in the clinic could provide treatment options for a vast array of malignancies.

## Conclusion

The discovery of Th17 cells has led to a plethora of studies targeting these cells to augment the antitumor response in patients. A number of basic findings have also advanced the cancer field through characterization of Th17 cells as a distinct subset that builds on the Th1/Th2 paradigm. As discussed herein, the role of Th17 cells in tumor immunity remains ambiguous and appears to be dependent upon several factors. Cytokines, costimulatory molecules and cell–cell interactions all impact the role of Th17 cells in the tumor milieu. While Th17 cells from human or murine tumors appear to favor the growth of a variety of malignancies by promoting angiogenesis or suppressing tumor immunity, convincing evidence demonstrates that adoptively transferred Th17 cells can mediate durable antitumor responses in mice with large tumors. However, the exact nature of how Th17 cells affect the course of tumor development remains poorly understood, in part because antigen-specificity of tumor-associated Th17 cells has not been defined in most cases.

Whether Th17 cells adopt a pro- or anti-tumorigenic role is largely dependent on the stimulation encountered by the cells. A better understanding of the signals that impact cell function and immunological fate could elucidate the driving force behind the Th17 identity crisis and is of considerable interest to the field of cancer therapy. Further studies on the manipulation of Th17 cells via blockades or genetic engineering for adoptive transfer are invaluable to the advancement of current therapies. More thorough knowledge of the mechanisms driving the antitumor response in these cells could lead to the development of enhanced vaccine and T cell-based therapies for patients with cancer.

## Conflict of Interest Statement

The authors declare that the research was conducted in the absence of any commercial or financial relationships that could be construed as a potential conflict of interest.

## References

[1] Muranski P Restifo NP Adoptive immunotherapy of cancer using CD4(+) T cells. Curr Opin Immunol (2009) 21(2):200–8 10.1016/j.coi.2009.02.004 19285848 PMC2715842

[2] Zhu J Yamane H Paul WE Differentiation of effector CD4 T cell populations. Annu Rev Immunol (2010) 28:445–89 10.1146/annurev-immunol-030409-101212 20192806 PMC3502616

[3] Zhu J Paul WE CD4 T cells: fates, functions, and faults. Blood (2008) 112(5):1557–69 10.1182/blood-2008-05-078154 18725574 PMC2518872

[4] Josefowicz SZ Lu LF Rudensky AY Regulatory T cells: mechanisms of differentiation and function. Annu Rev Immunol (2012) 30:531–64 10.1146/annurev.immunol.25.022106.141623 22224781 PMC6066374

[5] Mosmann TR Coffman RL TH1 and TH2 cells: different patterns of lymphokine secretion lead to different functional properties. Annu Rev Immunol (1989) 7:145–73 10.1146/annurev.iy.07.040189.001045 2523712

[6] Chamoto K Kosaka A Tsuji T Matsuzaki J Sato T Takeshima T Critical role of the Th1/Tc1 circuit for the generation of tumor-specific CTL during tumor eradication in vivo by Th1-cell therapy. Cancer Sci (2003) 94(10):924–8 10.1111/j.1349-7006.2003.tb01377.x 14556668 PMC11160164

[7] Ankathatti Munegowda M Xu S Freywald A Xiang J CD4+ Th2 cells function alike effector Tr1 and Th1 cells through the deletion of a single cytokine IL-6 and IL-10 gene. Mol Immunol (2012) 51(2):143–9 10.1016/j.molimm.2012.02.120 22424785

[8] Antony PA Restifo NP CD4+CD25+ T regulatory cells, immunotherapy of cancer, and interleukin-2. J Immunother (2005) 28(2):120–8 15725955 10.1097/01.cji.0000155049.26787.45 PMC1242172

[9] Turk MJ Guevara-Patino JA Rizzuto GA Engelhorn ME Houghton AN Concomitant tumor immunity to a poorly immunogenic melanoma is prevented by regulatory T cells. J Exp Med (2004) 200(6):771–82 10.1084/jem.20041130 15381730 PMC2211964

[10] Kidd P Th1/Th2 balance: the hypothesis, its limitations, and implications for health and disease. Altern Med Rev (2003) 8(3):233–46 12946237

[11] Steinman L A brief history of T(H)17, the first major revision in the T(H)1/T(H)2 hypothesis of T cell-mediated tissue damage. Nat Med (2007) 13(2):139–45 10.1038/nm0307-385a 17290272

[12] Zou W Restifo NP T(H)17 cells in tumor immunity and immunotherapy. Nat Rev Immunol (2010) 10:248–56 10.1038/nri2742 20336152 PMC3242804

[13] Martin F Apetoh L Ghiringhelli F Controversies on the role of Th17 in cancer: a TGF-beta-dependent immunosuppressive activity? Trends Mol Med (2012) 18(12):742–9 10.1016/j.molmed.2012.09.007 23083809

[14] Rizzo A De Mare V Rocchi C Stolfi C Colantoni A Neurath MF Smad7 induces plasticity in tumor-infiltrating Th17 cells and enable TNF-alpha-mediated killing of colorectal cancer cells. Carcinogenesis (2014). 10.1093/carcin/bgu027 24480808

[15] Korn T Bettelli E Oukka M Kuchroo VK IL-17 and Th17 cells. Annu Rev Immunol (2009) 27:485–517 10.1146/annurev.immunol.021908.132710 19132915

[16] Stritesky GL Yeh N Kaplan MH IL-23 promotes maintenance but not commitment to the Th17 lineage. J Immunol (2008) 181(9):5948–55 10.4049/jimmunol.181.9.5948 18941183 PMC2678905

[17] Liang SC Tan XY Luxenberg DP Karim R Dunussi-Joannopoulos K Collins M Interleukin (IL)-22 and IL-17 are coexpressed by Th17 cells and cooperatively enhance expression of antimicrobial peptides. J Exp Med (2006) 203(10):2271–9 10.1084/jem.20061308 16982811 PMC2118116

[18] Chang SH Dong C A novel heterodimeric cytokine consisting of IL-17 and IL-17F regulates inflammatory responses. Cell Res (2007) 17(5):435–40 17452998 10.1038/cr.2007.35

[19] Dong C TH17 cells in development: an updated view of their molecular identity and genetic programming. Nat Rev Immunol (2008) 8(5):337–48 10.1038/nri2295 18408735

[20] Ivanov II McKenzie BS Zhou L Tadokoro CE Lepelley A Lafaille JJ The orphan nuclear receptor RORgammat directs the differentiation program of proinflammatory IL-17+ T helper cells. Cell (2006) 126(6):1121–33 10.1016/j.cell.2006.07.035 16990136

[21] Ivanov II Zhou L Littman DR Transcriptional regulation of Th17 cell differentiation. Semin Immunol (2007) 19(6):409–17 10.1016/j.smim.2007.10.011 18053739 PMC2696342

[22] Veldhoen M Hirota K Westendorf AM Buer J Dumoutier L Renauld JC The aryl hydrocarbon receptor links TH17-cell-mediated autoimmunity to environmental toxins. Nature (2008) 453(7191):106–9 10.1038/nature06881 18362914

[23] Veldhoen M Hirota K Christensen J O’Garra A Stockinger B Natural agonists for aryl hydrocarbon receptor in culture medium are essential for optimal differentiation of Th17 T cells. J Exp Med (2009) 206(1):43–9 10.1084/jem.20081438 19114668 PMC2626686

[24] Ciofani M Madar A Galan C Sellars M Mace K Pauli F A validated regulatory network for Th17 cell specification. Cell (2012) 151(2):289–303 10.1016/j.cell.2012.09.016 23021777 PMC3503487

[25] Quintana FJ Basso AS Iglesias AH Korn T Farez MF Bettelli E Control of Treg and TH17 cell differentiation by the aryl hydrocarbon receptor. Nature (2008) 453:65–71 10.1038/nature06880 18362915

[26] Connor KT Aylward LL Human response to dioxin: aryl hydrocarbon receptor (AhR) molecular structure, function, and dose-response data for enzyme induction indicate an impaired human AhR. J Toxicol Environ Health B Crit Rev (2006) 9(2):147–71 10.1080/15287390500196487 16613807

[27] Rácz E Prens EP Kurek D Kant M de Ridder D Mourits S Effective treatment of psoriasis with narrow-band UVB phototherapy is linked to suppression of the IFN and Th17 pathways. J Invest Dermatol (2011) 131(7):1547–58 10.1038/jid.2011.53 21412260

[28] Furuhashi T Saito C Torii K Nishida E Yamazaki S Akimichi M Photo(chemo)therapy reduces circulating Th17 cells and restores circulating regulatory T cells in psoriasis. PLoS One (2013) 8(1):e54895 10.1371/journal.pone.0054895 23365685 PMC3554687

[29] Lo YH Torii K Saito C Furuhashi T Maeda A Morita A Serum IL-22 correlates with psoriatic severity and serum IL-6 correlates with susceptibiliy to phototherapy. J Dermatol Sci (2010) 58(3):225–7 10.1016/j.jdermsci.2010.03.018 20418068

[30] Ekman AK Sigurdardottir G Carlstrom M Kartul N Jenmalm MC Enerback C Systemically elevated Th1-, Th2- and Th17-associated chemokines in psoriasis vulgaris before and after ultraviolet B treatment. Acta Derm Venereol (2013) 93(5):527–31 10.2340/00015555-1545 23571825

[31] Bengsch B Seigel B Flecken T Wolanski J Blum HE Thimme R Human Th17 cells express high levels of enzymatically active dipeptidylpeptidase IV (CD26). J Immunol (2012) 188(11):5438–47 10.4049/jimmunol.1103801 22539793

[32] Mandapathil M Hilldorfer B Szczepanski MJ Czystowska M Szajnik M Ren J Generation and accumulation of immunosuppressive adenosine by human CD4+CD25highFOXP3+ regulatory T cells. J Biol Chem (2010) 285(10):7176–86 10.1074/jbc.M109.047423 19858205 PMC2844167

[33] Zhao Y Yang L Wang X Zhou Z The new insights of DPP-4 inhibitors: their potential immune modulatory function in autoimmune diabetes. Diabetes Metab Res Rev (2014). 10.1002/dmrr.2530 24446278

[34] Liu W Putnam AL Xu-Yu Z Szot GL Lee MR Zhu S CD127 expression inversely correlates with FoxP3 and suppressive function of human CD4+ T reg cells. J Exp Med (2006) 203(7):1701–11 10.1084/jem.20060772 16818678 PMC2118339

[35] Acosta-Rodriguez EV Rivino L Geginat J Jarrossay D Gattorno M Lanzavecchia A Surface phenotype and antigenic specificity of human interleukin 17-producing T helper memory cells. Nat Immunol (2007) 8(6):639–46 10.1038/ni1467 17486092

[36] Lee YK Turner H Maynard CL Oliver JR Chen D Elson CO Late developmental plasticity in the T helper 17 lineage. Immunity (2009) 30(1):92–107 10.1016/j.immuni.2008.11.005 19119024 PMC3607320

[37] Cosmi L De Palma R Santarlasci V Maggi L Capone M Frosali F Human interleukin 17-producing cells originate from a CD161+CD4+ T cell precursor. J Exp Med (2008) 205(8):1903–16 10.1084/jem.20080397 18663128 PMC2525581

[38] Wang Y Godec J Ben-Aissa K Cui K Zhao K Pucsek AB The transcription factors T-bet and Runx are required for the ontogeny of pathogenic interferon-γ-producing T helper 17 cell. Immunity (2014) 40(3):355–66 10.1016/j.immuni.2014.01.002 24530058 PMC3965587

[39] Kagami S Rizzo HL Lee JJ Koguchi Y Blauvelt A Circulating Th17, Th22, and Th1 cells are increased in psoriasis. J Invest Dermatol (2010) 130(5):1373–83 10.1038/jid.2009.399 20032993 PMC2892169

[40] Chen DY Chen YM Chen HH Hsieh CW Lin CC Lan JL Increasing levels of circulating Th17 cells and interleukin-17 in rheumatoid arthritis patients with an inadequate response to anti-TNF-alpha therapy. Arthritis Res Ther (2011) 13(4):R126 10.1186/ar3431 21801431 PMC3239366

[41] Zhang JP Yan J Xu J Pang XH Chen MS Li L Increased intratumoral IL-17-producing cells correlate with poor survival in hepatocellular carcinoma patients. J Hepatol (2009) 50(5):980–9 10.1016/j.jhep.2008.12.033 19329213

[42] Kesselring R Thiel A Pries R Trenkle T Wollenberg B Human Th17 cells can be induced through head and neck cancer and have a functional impact on HNSCC development. Br J Cancer (2010) 103(8):1245–54 10.1038/sj.bjc.6605891 20877351 PMC2967064

[43] Maruyama T Kono K Mizukami Y Kawaguchi Y Mimura K Watanabe M Distribution of Th17 cells and FoxP3(+) regulatory T cells in tumor-infiltrating lymphocytes, tumor-draining lymph nodes and peripheral blood lymphocytes in patients with gastric cancer. Cancer Sci (2010) 101(9):1947–54 10.1111/j.1349-7006.2010.01624.x 20550524 PMC11159855

[44] Tosolini M Kirilovsky A Mlecnik B Fredriksen T Mauger S Bindea G Clinical impact of different classes of infiltrating T cytotoxic and helper cells (Th1, th2, treg, th17) in patients with colorectal cancer. Cancer Res (2011) 71(4):1263–71 10.1158/0008-5472.CAN-10-2907 21303976

[45] Su X Ye J Hsueh EC Zhang Y Hoft DF Peng G Tumor microenvironments direct the recruitment and expansion of human Th17 cells. J Immunol (2010) 184(3):1630–41 10.4049/jimmunol.0902813 20026736

[46] Albert ML Sauter B Bhardwaj N Dendritic cells acquire antigen from apoptotic cells and induce class I-restricted CTLs. Nature (1998) 392(6671):86–9 10.1038/32183 9510252

[47] Kryczek I Banerjee M Cheng P Vatan L Szeliga W Wei S Phenotype, distribution, generation, and functional and clinical relevance of Th17 cells in the human tumor environments. Blood (2009) 114(6):1141–9 10.1182/blood-2009-03-208249 19470694 PMC2723011

[48] Derhovanessian E Adams V Hahnel K Groeger A Pandha H Ward S Pretreatment frequency of circulating IL-17+ CD4+ T-cells, but not Tregs, correlates with clinical response to whole-cell vaccination in prostate cancer patients. Int J Cancer (2009) 125(6):1372–9 10.1002/ijc.24497 19533748

[49] Chen X Wan J Liu J Xie W Diao X Xu J Increased IL-17-producing cells correlate with poor survival and lymphangiogenesis in NSCLC patients. Lung Cancer (2010) 69(3):348–54 10.1016/j.lungcan.2009.11.013 20022135

[50] Yang ZZ Novak AJ Ziesmer SC Witzig TE Ansell SM Malignant B cells skew the balance of regulatory T cells and TH17 cells in B-cell non-Hodgkin’s lymphoma. Cancer Res (2009) 69(13):5522–30 10.1158/0008-5472.CAN-09-0266 19509224 PMC2764404

[51] Horlock C Stott B Dyson PJ Morishita M Coombes RC Savage P The effects of trastuzumab on the CD4+CD25+FoxP3+ and CD4+IL17A+ T-cell axis in patients with breast cancer. Br J Cancer (2009) 100(7):1061–7 10.1038/sj.bjc.6604963 19277040 PMC2670001

[52] Wang W Edington HD Rao UN Jukic DM Radfar A Wang H Effects of high-dose IFNalpha2b on regional lymph node metastases of human melanoma: modulation of STAT5, FOXP3, and IL-17. Clin Cancer Res (2008) 14(24):8314–20 10.1158/1078-0432.CCR-08-0705 19088050

[53] von Euw E Chodon T Attar N Jalil J Koya RC Comin-Anduix B CTLA4 blockade increases Th17 cells in patients with metastatic melanoma. J Transl Med (2009) 7:35 10.1186/1479-5876-7-35 19457253 PMC2697137

[54] Dhodapkar KM Barbuto S Matthews P Kukreja A Mazumder A Vesole D Dendritic cells mediate the induction of polyfunctional human IL17-producing cells (Th17-1 cells) enriched in the bone marrow of patients with myeloma. Blood (2008) 112(7):2878–85 10.1182/blood-2008-03-143222 18669891 PMC2556623

[55] Zhou P Sha H Zhu J The role of T-helper 17 (Th17) cells in patients with medulloblastoma. J Int Med Res (2010) 38(2):611–9 10.1177/147323001003800223 20515574

[56] Li P Ji M Park J Bunting KD Ji C Tse W Th17 related cytokines in acute myeloid leukemia. Front Biosci (2012) 17:2284–94 10.2741/4052 22652779

[57] Sawada Y Nakamura M Kabashima-Kubo R Shimauchi T Kobayashi M Tokura Y Defective epidermal innate immunity and resultant superficial dermatophytosis in adult T-cell leukemia/lymphoma. Clin Cancer Res (2012) 18(14):3772–9 10.1158/1078-0432.CCR-12-0292 22648272

[58] Kuang DM Peng C Zhao Q Wu Y Chen MS Zheng L Activated monocytes in peritumoral stroma of hepatocellular carcinoma promote expansion of memory T helper 17 cells. Hepatology (2010) 51(1):154–64 10.1002/hep.23291 19902483

[59] Jain P Javdan M Feger FK Chiu PY Sison C Damle RN Th17 and non-Th17 interleukin-17-expressing cells in chronic lymphocytic leukemia: delineation, distribution, and clinical relevance. Haematologica (2012) 97(4):599–607 10.3324/haematol.2011.047316 22058222 PMC3347674

[60] Wu S Rhee KJ Albesiano E Rabizadeh S Wu X Yen HR A human colonic commensal promotes colon tumorigenesis via activation of T helper type 17 T cell responses. Nat Med (2009) 15(9):1016–22 10.1038/nm.2015 19701202 PMC3034219

[61] Wick EC Rabizadeh S Albesiano E Wu X Wu S Chan J Stat3 activation in murine colitis induced by enterotoxigenic *Bacteroides fragilis*. Inflamm Bowel Dis (2014) 20(5):821–34 10.1097/MIB.0000000000000019 24704822 PMC4121853

[62] Paulos CM Wrzesinski C Kaiser A Hinrichs CS Chieppa M Cassard L Microbial translocation augments the function of adoptively transferred self/tumor-specific CD8+ T cells via TLR4 signaling. J Clin Invest (2007) 117(8):2197–204 10.1172/JCI32205C1 17657310 PMC1924500

[63] He D Li H Yusuf N Elmets CA Athar M Katiyar SK IL-17 mediated inflammation promotes tumor growth and progression in the skin. PLoS One (2012) 7(2):e32126 10.1371/journal.pone.0032126 22359662 PMC3281112

[64] Wang L Yi T Zhang W Pardoll DM Yu H IL-17 enhances tumor development in carcinogen-induced skin cancer. Cancer Res (2010) 70(24):10112–20 10.1158/0008-5472 21159633 PMC3059780

[65] Muranski P Boni A Antony PA Cassard L Irvine KR Kaiser A Tumor-specific Th17-polarized cells eradicate large established melanoma. Blood (2008) 112(2):362–73 10.1182/blood-2007-11-120998 18354038 PMC2442746

[66] Martin-Orozco N Muranski P Chung Y Yang XO Yamazaki T Lu S T helper 17 cells promote cytotoxic T cell activation in tumor immunity. Immunity (2009) 31(5):787–98 10.1016/j.immuni.2009.09.014 19879162 PMC2787786

[67] Demaria S Pikarsky E Karin M Coussens LM Chen YC El-Omar EM Cancer and inflammation: promise for biologic therapy. J Immunother (2010) 33(4):335–51 10.1097/CJI.0b013e3181d32e74 20386472 PMC2941912

[68] DeNardo DG Coussens LM Inflammation and breast cancer. Balancing immune response: crosstalk between adaptive and innate immune cells during breast cancer progression. Breast Cancer Res (2007) 9(4):212 10.1186/bcr1746 17705880 PMC2206719

[69] Tan TT Coussens LM Humoral immunity, inflammation and cancer. Curr Opin Immunol (2007) 19(2):209–16 10.1016/j.coi.2007.01.001 17276050

[70] van Kempen LC de Visser KE Coussens LM Inflammation, proteases and cancer. Eur J Cancer (2006) 42(6):728–34 10.1016/j.ejca.2006.01.004 16524717

[71] Coussens LM Werb Z Inflammation and cancer. Nature (2002) 420(6917):860–7 10.1038/nature01322 12490959 PMC2803035

[72] Lanca T Silva-Santos B The split nature of tumor-infiltrating leukocytes: implications for cancer surveillance and immunotherapy. Oncoimmunology (2012) 1(5):717–25 10.4161/onci.20068 22934263 PMC3429575

[73] Dai Y Jiao H Teng G Wang W Zhang R Wang Y Embelin reduces colitis-associated tumorigenesis through limiting IL-6/STAT3 signaling. Mol Cancer Ther (2014) 13(5):1206–16 10.1158/1535-7163.MCT-13-0378 24651526

[74] Jochems C Schlom J Tumor-infiltrating immune cells and prognosis: the potential link between conventional cancer therapy and immunity. Exp Biol Med (Maywood) (2011) 236(5):567–79 10.1258/ebm.2011.011007 21486861 PMC3229261

[75] Bremnes RM Al-Shibli K Donnem T Sirera R Al-Saad S Andersen S The role of tumor-infiltrating immune cells and chronic inflammation at the tumor site on cancer development, progression, and prognosis: emphasis on non-small cell lung cancer. J Thorac Oncol (2011) 6(4):824–33 10.1097/JTO.0b013e3182037b76 21173711

[76] Fialová A Partlová S Sojka L Hromádková H Brtnický T Fucíková J Dynamics of T-cell infiltration during the course of ovarian cancer: the gradual shift from a Th17 effector cell response to a predominant infiltration by regulatory T-cells. Int J Cancer (2013) 132(5):1070–9 10.1002/ijc.27759 22865582

[77] Winkler I Gogacz M Rechberger T Do Th17 cells play an important role in the pathogenesis and prognosis of ovarian cancer? Ginekol Pol (2012) 83(4):295–300 22712263

[78] Munn DH Th17 cells in ovarian cancer. Blood (2009) 114(6):1134–5 10.1182/blood-2009-06-224246 19661273

[79] Kim JS Sklarz T Banks LB Gohil M Waickman AT Skuli N Natural and inducible TH17 cells are regulated differently by Akt and mTOR pathways. Nat Immunol (2013) 14(6):611–8 10.1038/ni.2607 23644504 PMC3711189

[80] Candela M Turroni S Biagi E Carbonero F Rampelli S Fiorentini C Inflammation and colorectal cancer, when microbiota-host mutualism breaks. World J Gastroenterol (2014) 20(4):908–22 10.3748/wjg.v20.i4.908 24574765 PMC3921544

[81] Baumgart S Chen NM Siveke JT König A Zhang JS Singh SK Inflammation-induced NFATc1-STAT3 transcription complex promotes pancreatic cancer initiation by KrasG12D. Cancer Discov (2014) 4(6):688–701 10.1158/2159-8290.CD-13-0593 24694735 PMC4069603

[82] Rielland M Cantor DJ Graveline R Hajdu C Mara L de Diego Diaz B Senescence-associated SIN3B promotes inflammation and pancreatic cancer progression. J Clin Invest (2014) 124(5):2125–35 10.1172/JCI72619 24691445 PMC4001548

[83] Pinato DJ Shiner RJ Seckl MJ Stebbing J Sharma R Mauri FA Prognostic performance of inflammation-based prognostic indices in primary operable non-small cell lung cancer. Br J Cancer (2014) 110(8):1930–5 10.1038/bjc.2014.145 24667648 PMC3992503

[84] Keerthivasan S Aghajani K Dose M Molinero L Khan MW Venkateswaran V β-Catenin promotes colitis and colon cancer through imprinting of proinflammatory properties in T cells. Sci Transl Med (2014) 6(225):225ra28 10.1126/scitranslmed.3007607 24574339 PMC4020714

[85] Su Z Sun Y Zhu H Liu Y Lin X Shen H Th17 cell expansion in gastric cancer may contribute to cancer development and metastasis. Immunol Res (2014) 58(1):118–24 10.1007/s12026-013-8483-y 24402773

[86] Karin M Lawrence T Nizet V Innate immunity gone awry: linking microbial infections to chronic inflammation and cancer. Cell (2006) 124(4):823–35 10.1016/j.cell.2006.02.016 16497591

[87] Numasaki M Fukushi J Ono M Narula SK Zavodny PJ Kudo T Interleukin-17 promotes angiogenesis and tumor growth. Blood (2003) 101(7):2620–7 10.1182/blood-2002-05-1461 12411307

[88] Chang SH Mirabolfathinejad SG Katta H Cumpian AM Gong L Caetano MS T helper 17 cells play a critical pathogenic role in lung cancer. Proc Natl Acad Sci U S A (2014) 111(15):5664–9 10.1073/pnas.1319051111 24706787 PMC3992670

[89] Kryczek I Wei S Szeliga W Vatan L Zou W Endogenous IL-17 contributes to reduced tumor growth and metastasis. Blood (2009) 114(2):357–9 10.1182/blood-2008-09-177360 19289853 PMC2714210

[90] Zhang YL Li J Mo HY Qui F Zheng LM Qian CN Different subsets of tumor infiltrating lymphocytes correlate with NPC progression in different ways. Mol Cancer (2010) 9:4 10.1186/1476-4598-9-4 20064222 PMC2818695

[91] Tong Z Yang XO Yan H Liu W Niu X Shi Y A protective role by interleukin-17F in colon tumorigenesis. PLoS One (2012) 7(4):e34959 10.1371/journal.pone.0034959 22509371 PMC3324558

[92] Weber GF Gaertner FC Erl W Janssen KP Blechert B Holzmann B IL-22-mediated tumor growth reduction correlates with inhibition of ERK1/2 and AKT phosphorylation and induction of cell cycle arrest in the G2-M phase. J Immunol (2006) 177(11):8266–72 10.4049/jimmunol.177.11.8266 17114505

[93] Castermans K Tabruyn SP Zeng R van Beijnum JR Eppolito C Leonard WJ Angiostatic activity of the antitumor cytokine interleukin-21. Blood (2008) 112(13):4940–7 10.1182/blood-2007-09-113878 18515660 PMC6561391

[94] Zielinski CE Mele F Aschenbrenner D Jarrossay D Ronchi F Gattorno M Pathogen-induced human TH17 cells produce IFN-gamma or IL-10 and are regulated by IL-1beta. Nature (2012) 484(7395):514–8 10.1038/nature10957 22466287

[95] Xu X Zheng S Yang F Shi Y Gu Y Chen H Increased Th22 cells are independently associated with Th17 cells in type 1 diabetes. Endocrine (2014) 46(1):90–8 10.1007/s12020-013-0030-z 23928796

[96] Kobold S Völk S Clauditz T Küpper NJ Minner S Tufman A Interleukin-22 is frequently expressed in small- and large-cell lung cancer and promotes growth in chemotherapy-resistant cancer cells. J Thorac Oncol (2013) 8(8):1032–42 10.1097/JTO.0b013e31829923c8 23774470

[97] Weaver CT Harrington LE Mangan PR Gavrieli M Murphy KM Th17: an effector CD4 T cell lineage with regulatory T cell ties. Immunity (2006) 24(6):677–88 10.1016/j.immuni.2006.06.002 16782025

[98] Gomez-Rodriguez J Wohlfert EA Handon R Meylan F Wu JZ Anderson SM Itk-mediated integration of T cell receptor and cytokine signaling regulates the balance between Th17 and regulatory T cells. J Exp Med (2014) 211(3):529–43 10.1084/jem.20131459 24534190 PMC3949578

[99] Chalmin F Mignot G Bruchard M Chevriaux A Végran F Hichami A Stat3 and Gfi-1 transcription factors control Th17 cell immunosuppressive activity via the regulation of ectonucleotidase expression. Immunity (2012) 36(3):362–73 10.1016/j.immuni.2011.12.019 22406269

[100] Valmori D Raffin C Raimbaud I Ayyoub M Human RORgammat+ TH17 cells preferentially differentiate from naive FOXP3+Treg in the presence of lineage-specific polarizing factors. Proc Natl Acad Sci U S A (2010) 107(45):19402–7 10.1073/pnas.1008247107 20962281 PMC2984184

[101] Koenen HJ Smeets RL Vink PM van Rijssen E Boots AM Joosten I Human CD25highFoxp3pos regulatory T cells differentiate into IL-17-producing cells. Blood (2008) 112(6):2340–52 10.1182/blood-2008-01-133967 18617638

[102] Noack M Miossec P Th17 and regulatory T cell balance in autoimmune and inflammatory diseases. Autoimmun Rev (2014) 13(6):668–77 10.1016/j.autrev.2013.12.004 24418308

[103] Du J Huang C Zhou B Ziegler SF Isoform-specific inhibition of ROR alpha-mediated transcriptional activation by human FOXP3. J Immunol (2008) 180(7):4785–92 10.4049/jimmunol.180.7.4785 18354202

[104] Tartar DM VanMorlan AM Wan X Guloglu FB Jain R Haymaker CL FoxP3+RORgammat+ T helper intermediates display suppressive function against autoimmune diabetes. J Immunol (2010) 184(7):3377–85 10.4049/jimmunol.0903324 20181889 PMC2843758

[105] McGeachy MJ Bak-Jensen KS Chen Y Tato CM Blumenschein W McClanahan T TGF-beta and IL-6 drive the production of IL-17 and IL-10 by T cells and restrain T(H)-17 cell-mediated pathology. Nat Immunol (2007) 8(12):1390–7 10.1038/ni1539 17994024

[106] Ghoreschi K Laurence A Yang XP Tato CM McGeachy MJ Konkel JE Generation of pathogenic T(H)17 cells in the absence of TGF-beta signalling. Nature (2010) 467(7318):967–71 10.1038/nature09447 20962846 PMC3108066

[107] Muranski P Borman ZA Kerkar SP Klebanoff CA Ji Y Sanchez-Perez L Th17 cells are long lived and retain a stem cell-like molecular signature. Immunity (2011) 35(6):972–85 10.1016/j.immuni.2011.09.019 22177921 PMC3246082

[108] Wu C Yosef N Thalhamer T Zhu C Xiao S Kishi Y Induction of pathogenic Th17 cells by inducible salt sensing kinase SGK1. Nature (2013) 496(7446):513–7 10.1038/nature11984 23467085 PMC3637879

[109] Lee Y Awasthi A Yosef N Quintana FJ Xiao S Peters A Induction and molecular signature of pathogenic TH17 cells. Nat Immunol (2012) 13(10):991–9 10.1038/ni.2416 22961052 PMC3459594

[110] Porter DL Levine BL Kalos M Bagg A June CH Chimeric antigen receptor-modified T cells in chronic lymphoid leukemia. N Engl J Med (2011) 365:725–33 10.1056/NEJMoa1103849 21830940 PMC3387277

[111] Restifo NP Dudley ME Rosenberg SA Adoptive immunotherapy for cancer: harnessing the T cell response. Nat Rev Immunol (2012) 12(4):269–81 10.1038/nri3191 22437939 PMC6292222

[112] Robbins PF Morgan RA Feldman SA Yang JC Sherry RM Dudley ME Tumor regression in patients with metastatic synovial cell sarcoma and melanoma using genetically engineered lymphocytes reactive with NY-ESO-1. J Clin Oncol (2011) 29(7):917–24 10.1200/JCO.2010.32.2537 21282551 PMC3068063

[113] Paulos CM Suhoski MM Plesa G Jiang T Basu S Golovina TN Adoptive immunotherapy: good habits instilled at youth have long-term benefits. Immunol Res (2008) 42(1–3):182–96 10.1007/s12026-008-8070-9 18949448 PMC3809041

[114] Luckey CJ Weaver CT Stem-cell-like qualities of immune memory; CD4+ T cells join the party. Cell Stem Cell (2012) 10(2):107–8 10.1016/j.stem.2012.01.011 22305557 PMC3277283

[115] Gattinoni L Zhong XS Palmer DC Ji Y Hinrichs CS Yu Z Wnt signaling arrests effector T cell differentiation and generates CD8+ memory stem cells. Nat Med (2009) 15(7):808–13 10.1038/nm.1982 19525962 PMC2707501

[116] Kryczek I Zhao E Liu Y Wang Y Vatan L Szeliga W Human TH17 cells are long-lived effector memory cells. Sci Transl Med (2011) 3(104):104ra100 10.1126/scitranslmed.3002949 21998407 PMC3345568

[117] Yu Y Iclozan C Yamazaki T Yang X Anasetti C Dong C Abundant c-Fas-associated death domain-like interleukin-1-converting enzyme inhibitory protein expression determines resistance of T helper 17 cells to activation-induced cell death. Blood (2009) 114(5):1026–8 10.1182/blood-2009-03-210153 19429865 PMC2721783

[118] Ankathatti Munegowda M Deng Y Mulligan SJ Xiang J Th17 and Th17-stimulated CD8+ T cells play a distinct role in Th17-induced preventative and therapeutic antitumor immunity. Cancer Immunol Immunother (2011) 60(10):1473–84 10.1007/s00262-011-1054-y 21660450 PMC11028972

[119] Xie Y Akpinarli A Maris C Hipkiss EL Lane M Kwon EK Naive tumor-specific CD4(+) T cells differentiated in vivo eradicate established melanoma. J Exp Med (2010) 207(3):651–67 10.1084/jem.20091921 20156973 PMC2839147

[120] Kryczek I Wei S Zou L Altuwaijri S Szeliga W Kolls J Cutting edge: Th17 and regulatory T cell dynamics and the regulation by IL-2 in the tumor microenvironment. J Immunol (2007) 178(11):6730–3 10.4049/jimmunol.178.11.6730 17513719

[121] Tai X Erman B Alag A Mu J Kimura M Katz G Foxp3 transcription factor is proapoptotic and lethal to developing regulatory T cells unless counterbalanced by cytokine survival signals. Immunity (2013) 38(6):1116–28 10.1016/j.immuni.2013.02.022 23746651 PMC3700677

[122] Chen Y Haines CJ Gutcher I Hochweller K Blumenschein WM McClanahan T Foxp3(+) regulatory T cells promote T helper 17 cell development in vivo through regulation of interleukin-2. Immunity (2011) 34(3):409–21 10.1016/j.immuni.2011.02.011 21435588

[123] Roychoudhuri R Hirahara K Mousavi K Clever D Klebanoff CA Bonelli M BACH2 represses effector programs to stabilize T(reg)-mediated immune homeostasis. Nature (2013) 498(7455):506–10 10.1038/nature12199 23728300 PMC3710737

[124] Muto A Tashiro S Nakajima O Hoshino H Takahashi S Sakoda E The transcriptional programme of antibody class switching involves the repressor Bach2. Nature (2004) 429(6991):566–71 10.1038/nature02596 15152264

[125] Marsh JL Jackman CP Tang S Shankar S Srivastava RK Embelin suppresses pancreatic cancer growth by modulating tumor immune microenvironment. Front Biosci (2014) 19:113–25 10.2741/4198 24389175

[126] June CH Adoptive T cell therapy for cancer in the clinic. J Clin Invest (2007) 117(6):1466–76 10.1172/JCI32446 17549249 PMC1878537

[127] Gattinoni L Powell DJ Jr Rosenberg SA Restifo NP Adoptive immunotherapy for cancer: building on success. Nat Rev Immunol (2006) 6(5):383–93 10.1038/nri1842 16622476 PMC1473162

[128] Paulos CM Carpenito C Plesa G Suhoski MM Varela-Rohena A Golovina TN The inducible costimulator (ICOS) is critical for the development of human T(H)17 cells. Sci Transl Med (2010) 2(55):55ra78 10.1126/scitranslmed.3000448 20980695 PMC6282816

[129] Garaude J Blander JM ICOStomizing immunotherapies with T(H)17. Sci Transl Med (2010) 2(55):55pa52 10.1126/scitranslmed.3001722 20980694

[130] Varela-Rohena A Carpenito C Perez EE Richardson M Parry RV Milone M Genetic engineering of T cells for adoptive immunotherapy. Immunol Res (2008) 42(1–3):166–81 10.1007/s12026-008-8057-6 18841331 PMC2699549

[131] Zhao Y Moon E Carpenito C Paulos CM Liu X Brennan AL Multiple injections of electroporated autologous T cells expressing a chimeric antigen receptor mediate regression of human disseminated tumor. Cancer Res (2010) 70(22):9053–61 10.1158/0008-5472.CAN-10-2880 20926399 PMC2982929

[132] Kalos M Levine BL Porter DL Katz S Grupp SA Bagg A T cells with chimeric antigen receptors have potent antitumor effects and can establish memory in patients with advanced leukemia. Sci Transl Med (2011) 3(95):95ra73 10.1126/scitranslmed.3002842 21832238 PMC3393096

[133] Liddy N Bossi G Adams KJ Lissina A Mahon TM Hassan NJ Monoclonal TCR-redirected tumor cell killing. Nat Med (2012) 18(6):980–7 10.1038/nm.2764 22561687

[134] Milone MC Fish JD Carpenito C Carroll RG Binder GK Teachey D Chimeric receptors containing CD137 signal transduction domains mediate enhanced survival of T cells and increased antileukemic efficacy in vivo. Mol Ther (2009) 17(8):1453–64 10.1038/mt.2009.83 19384291 PMC2805264

[135] Al-Khami AA Mehrotra S Nishimura MI Adoptive immunotherapy of cancer: gene transfer of T cell specificity. Self Nonself (2011) 2(2):80–4 10.4161/self.2.2.15832 22299059 PMC3268993

[136] Gattinoni L Klebanoff CA Palmer DC Wrzesinski C Kerstann K Yu Z Acquisition of full effector function in vitro paradoxically impairs the in vivo antitumor efficacy of adoptively transferred CD8+ T cells. J Clin Invest (2005) 115(6):1616–26 15931392 10.1172/JCI24480 PMC1137001

